# Instrumental support: A conceptual analysis

**DOI:** 10.1111/nuf.12704

**Published:** 2022-02-08

**Authors:** Beth E. Schultz, Cynthia F. Corbett, Ronda G. Hughes

**Affiliations:** ^1^ College of Pharmacy, Natural & Health Sciences Manchester University Fort Wayne Indiana USA; ^2^ College of Nursing University of South Carolina Columbia South Carolina USA

**Keywords:** care transitions, concept analysis, instrumental support, social support

## Abstract

The aim of this paper was to use the Walker and Avant method of concept analysis to evaluate the concept of instrumental support in the context of hospital to home care transitions. Findings from this concept analysis suggest three defining attributes of instrumental support: informal support providers, tangible support, and unmet personal needs. Antecedents identified: a strong and supportive social network, an independently functioning adult, an illness resulting in hospitalization, a change in functional status, and the patient being discharged home from the hospital. Consequences of not having adequate instrumental support: unsuccessful recovery at home, increased risk for hospital readmission, decline in physical functioning, health complications, and increased risk of mortality. Empirical referents: patient's report of successful recovery, returning to an independent level of functioning, and the lack of hospital readmission or health complication. A model and a contrary case study were developed to provide examples of clinical cases related to instrumental support. Recommendations related to clinical practice include evaluating the availability/adequacy of instrumental support before hospital discharge and including the identified instrumental support person in the discharge planning process. There are proven benefits of having people within one's social network providing instrumental support during the home recovery period.

## 1 INTRODUCTION

The knowledge that people need people to thrive is well supported in research.^1^, ^2^ The presence of adequate, effective social support is linked to both physical and mental well‐being and has been the focus of researchers in both behavioral and health sciences. As one ages, there are times when one needs assistance from an informal network of friends, neighbors, and family,^3^ for example, during a time of illness and recovery. This informal support system provides help during times of need in the form of social interaction and can assist with tasks such as transportation and meal preparation. Assistance provided to meet tangible needs is known as instrumental support, a type of social support.^4^ Instrumental support is identified by actions that include help with personal and medical care, transportation, and meal preparation.^5^

Patients are discharged from the hospital with the expectation that recovery will take place in the home setting. During the critical time after discharge older patients experience increased fatigue, lack of energy and motivation, decreased muscle strength, and decreased coordination^6^; these symptoms are linked to the effects of hospitalization.^7^ The transient hospital to home period often creates a need for assistance after discharge for previously independent older adults because hospital experiences may include disturbed sleep patterns, inadequate nutritional intake, pain, mental stress and fatigue, and information overload that can negatively impact recovery. Krumholz coined this phenomenon “posthospital syndrome.” Patients are discharged home with less stamina and in a weakened state and are often unable to take care of their own basic needs.^8^ Regaining one's preadmission level of functioning and independence takes time and the effects of posthospital syndrome may persist up to 3 months after discharge, creating the need for help at home from family and friends.^9^

Recent research has shown that the lack of people to provide assistance after illness inhibits a patient's ability to successfully recover at home.^10^, ^11^, ^12^ Patients have commonly identified a lack of help after discharge as a factor that led to readmission, and thus an unsuccessful care transition experience. Understanding needs post discharge, including the availability of and effectiveness of instrumental support, and the impact the support has on the patient's recovery, leads to a need to evaluate the concept of instrumental support. The relationship between the availability and adequacy of help at home after discharge and whether the patient experiences readmission is of great interest and is the focus of this concept analysis. This interest leads to the question, “Does having adequate instrumental support at home after discharge decrease the incidence of readmission?”. An important step in answering this question is understanding the concept of instrumental support. Reviewers followed an established process developed by Walker and Avant to evaluate the concept's structure and function in the context of the literature and practice.^13^ Ultimately, the goal is to positively impact patient outcomes related to hospital to home care transitions.

## 2 ORIGINS OF THE CONCEPT

The term “instrumental” has a Medieval Latin origin meaning “a tool or implement,” and the literal meaning from the breakdown of the word parts is “to build.”^14^ The Merriam‐Webster Dictionary^15^ includes the definition “serving as a crucial means.” Tabers Medical Dictionary defines instrumental as “important in achieving a result or goal.”^16^ A consistent theme found in these definitions is the idea of bringing about change. The definitions indicate the implements or the process of transformation.

A review of the literature revealed that the concept of instrumental support was introduced by behavioral scientists and was used to describe a subcategory of social support, along with other terms that focused on the social needs of individuals.^4^ The concept is also used in research evaluating social support in the workplace, discussing aid received when performing tasks.^17^, ^18^ Most recently, Usman et al. evaluated the impact of instrumental support in the healthcare work environment and its impact on managing emotional exhaustion. Other terms used interchangeably with instrumental support include tangible, functional, informal, practical, enacted, and task support. These terms are not used consistently, but are used in the same context in the literature. In some cases, the act of providing assistance to/by family and friends was described, but not specifically labeled, which made the synthesis of the concept challenging. However, instrumental support is the most frequent term used.

## 3 METHODS

For this concept analysis, literature from various disciplines that study instrumental support was evaluated. We wanted a broad range of uses to understand the concept and determine whether its meaning was consistent across disciplines. Databases searched were PubMed, CINAHL, APA PsychInfo, and MEDLINE using the term “instrumental support.” The publications were filtered for adults, English language, and academic publications. No limit was placed on the year of publication. The initial search returned 886 publications. The titles were screened with 804 articles eliminated. Following the abstract review, 30 articles published between 1997 and 2021 were selected for inclusion in this concept analysis of instrumental support.

## 4 FINDINGS

### 4.1 Defining attributes

Defining attributes are elements that consistently appear in the literature when the concept is presented or discussed.^13^ The defining attributes of instrumental support are informal support providers, tangible support, and unmet personal needs.

#### 4.1.1 Informal support

The attribute of informal support was identified as a fundamental characteristic of instrumental support in the context of help. “Informal” indicated that the care was not being provided by a paid provider, for example, someone from a home health agency. Of the 30 articles used to evaluate the concept, 15 of them specifically provided the context of who provided assistance. In nine of the articles, “family” or “family member” was mentioned.^10^, ^19^, ^20^, ^21^, ^22^, ^23^, ^24^, ^25^, ^26^, ^27^ The subject of the articles varied from managing stressful life situations,^24^, ^27^ to readmissions.^20^ Articles provided perspectives from both the patients^10^ and family members.^19^ The phrase “someone to look after you” was used instead of identifying a familial or social connection in the context of improving psychological wellbeing^28^ and decreasing the risk of readmission.^29^ “Friend” was also used to describe the person who would help with health care and medications,^21^, ^27^ be present during meetings to talk about discharge planning,^30^ or help with managing chronic illness.^25^ The term “caregiver” was used in combination with friends and/or family, but was also used alone without any defining characteristics. The term “caregivers” was used in the context of readmission^12^ and also for the person providing care for functionally or physically impaired adults.^27^, ^31^ Researchers evaluating instrumental support in the workplace identified providers as “coworkers.”^17^, ^18^ Research is emerging linking the positive impact instrumental support has in the healthcare setting related to the stress healthcare providers are experiencing related to the pandemic.^18^, ^32^ Having people within one's social circle or in the workplace who are willing to provide help during a time of need can positively impact a person's physical and mental health.^33^

#### 4.1.2 Anticipated tangible support needs

The attribute “tangible support” indicates actionable help that meets specific needs. People may be unable to drive or use public transportation as a result of an illness or a recent short‐term physical limitation that impacts their ability to attend follow‐up appointments,^34^ pick‐up medications,^10^ or go grocery shopping.^35^ Because of the effects of illness people may lack the energy they need to complete personal care tasks,^36^ clean their home,^37^ or prepare nutritionally appropriate meals.^25^ Home environments may need to be modified ^36^ for people who experience impaired mobility and may be at an increased risk for falls.^8^ Family and friends who anticipate the needs a patient will have after discharge may develop a plan to provide assistance, prepare meals, or help them with other tasks once the patient returns home.

#### 4.1.3 Identified unmet needs

Patients often experience the defining attribute “identified unmet needs” after an illness. There are times when the needs are anticipated and can be addressed before discharge, and other times when the need becomes evident after the patient has returned home. People who are normally independent, use public transportation, or drive themselves may experience restrictions related to driving or ambulation after illness,^19^ and may become dependent on others for transportation.^8^ Another characteristic of this attribute is the need for help with personal care. Some researchers reported that patients were unable to bath and dress without help,^38^ or were unable to complete personal tasks and meet their own basic needs,^25^, ^26^ or that caregivers reported they were unable to transfer patients who have experienced a stroke.^19^ Other researchers found that patients were also unable to complete basic housekeeping tasks,^23^, ^26^, ^37^ prepare meals, or were unable to get groceries because they were fatigued.^19^, ^26^, ^36^ Additionally, some researchers discovered patients had unmet needs related to disease and medication management.^10^, ^19^, ^21^, ^38^ These types of unmet needs, lack of transportation, inability to provide self‐care, prepare meals, or self‐manage health conditions contributes to hospital readmission.^20^, ^21^

### 4.2 Antecedents

Antecedents are events or circumstances that must occur before the concept can occur.^39^ An important antecedent for instrumental support is a strong, supportive social network in which those within the social network provide and receive help when needed. The social network could include family and/or friends. In the context of care transitions, an active, independent adult must experience an admission as the result of the onset or exacerbation of an illness/injury.^19^, ^25^, ^27^ Another antecedent is that there must be a care transition from the hospital to home setting,^36^, ^40^ and finally, the patient experiences a change in functional status and does not return home with the same degree of independence they have before the event.^7^, ^8^

### 4.3 Consequences

Consequences are the events that occur as the result of the occurrence of the concept.^39^ The consequence of instrumental support depends on whether the patient has a social or family network to meet their needs after discharge. If that patient has family and friends providing informal support that adequately meets their identified needs, the result is likely to be a successful recovery at home after discharge.^25^, ^41^ A negative consequence of the lack of supportive friends or family to help during the time a patient is recovering at home can be an increase in readmissions and/or continual decline in physical functioning,^42^ illness‐related complications,^11^ or increased risk of mortality.^41^

### 4.4 Empirical referents

Empirical referents are attributes that demonstrate the concept itself.^39^ The patient's report of appropriate support from family and/or friends who aided them in the recovery process to meet personal needs is anecdotal evidence of effective instrumental support. Successful management of chronic diseases,^40^ returning to an independent level of functioning,^9^ and the lack of hospital readmission^43^ can also be used to evaluate whether the patient successfully recovered at home.

### 4.5 Model case

Eleanor McPhail was a 77‐year‐old widowed woman who lives alone. She has two grown children, the nearest one lives more than an hour away. One of her children is working in South Asia and unable to come home and the other recently had a hip replacement. She has long‐term neighbors and friends she socializes with on a regular basis and attends a local church. Eleanor is not feeling well and makes an appointment with her primary care provider. She is diagnosed with pneumonia and is admitted to the hospital. After several days, she is ready to be discharged home. She tells the nurse that she has plenty of friends who will help her until she gets back on her feet. Eleanor talks with her children who are concerned about her going home and she tells them not to worry and that her friends will help her. Two of her neighbors come to pick her up and drive her and her car back home. She is weak, experiences fatigue when she is up and around, and feels like she has a bad case of jetlag. Her friends create a schedule to check on her every day; one friend who is a retired nurse helps her bathe, while others prepare meals that she can warm in the microwave and drive her to appointments. One friend that she walks with each day sets up short‐term goals to promote a gradual increase in Eleanor's physical activity. Eleanor slowly regains her strength and stamina. During her follow‐up visit with her primary care provider, she shares that without her friends she would not be doing as well as she is. Within 6 weeks Eleanor is taking care of her own needs and is able to walk a little further every day. With the support of her friends, she is able to successfully recover at home and regain her independence.

### 4.6 Contrary case

Richard is an 81‐year‐old widower who lives alone. He moved to his current home after retirement with his wife who died 3 years ago. The neighbors they knew had all moved out of the area and Richard stopped going to church after his wife died. He does go to the local AmVets for a drink and game night once a week. He no longer drives, but he can use public transportation when needed and can walk to get groceries, go to the library, see his healthcare provider, and go to the barber. He has one son that he talks with occasionally, but his son lives 2 hours away. Richard is experiencing shortness of breath and is unable to sleep because of coughing. After trying home remedies that usually work and not getting any better, he walks over to the local clinic to be seen. Richard is given prescriptions for antibiotics and steroids and an appointment to return to the clinic in a week. He walks to the pharmacy to get his prescriptions, stops at the grocery store, and picks up food that does not require much preparation and returns home. He uses his “Alexa” to set times for when he is supposed to take his medicine and remind him to drink water and get up and walk around his apartment. Over the course of the week, Richard takes his medication, eats sandwiches because he does not have the energy to cook, bathes at the sink because he is unsteady on his feet, and stays inside because he does not feel like walking to the library. Someone he knows from the AmVets called when he did not show up for a game night and Richard tells them he is sick and cannot get out. They tell him they hope he feels better and will be able to attend next week. When he returns to the clinic his lungs sound better, but he is much weaker, and the healthcare provider notes he is unsteady. He is concerned when Richard reports that he is not able to engage in his normal activities, get into the shower or prepare a hot meal. Based on his report of the lack of a social network, his healthcare provider asks him to consider moving closer to family.

## 5 IMPLICATIONS FOR CLINICAL PRACTICE

The time of transition from hospital to home is a critical time with the potential for complications. The combination of disturbed sleep patterns, mental confusion, fatigue, and other effects of hospitalization increase a patient's risk for complications and readmission.^7^ During discharge planning, healthcare providers should assess a patient's availability of postdischarge support from family and/or friends to help them with personal and medical care to determine if they will have the help they need.^44^ In addition to patient's perspectives of available postdischarge support, research shows that the input from the nurse is extremely important.^45^ Nurses observe interactions between patients and family/friends and may have an idea if they will be able to meet the patient's needs while they recover at home. Having someone present is important but making sure the patient's needs are met is essential to a successful transition. Including the people who will be involved in providing care after discharge may lead to a safer transition from hospital to home.^43^ Support persons should be provided with information about what the patient's needs will be, what the expectations are of a caregiver, and what to expect during the recovery process. Having these processes in place has the potential of decreasing complications and hospital readmissions and increasing patients' and caregivers' feeling of readiness for discharge and care management at home.^46^

## 6 CONCLUSIONS

The foundational implication of the concept of instrumental support is that people often need others present to help them successfully recover after an illness and regain independence. The knowledge of whether family and friends are available to provide hands‐on assistance is important when planning hospital to home transitions. Determining whether the caregivers will “be present” and able to meet the person's needs should be discerned by including the patient and caregiver in the discharge process. Older adults especially are impacted by the effects of posthospital syndrome and require people who are willing to be intentional about providing assistance to meet their needs.

As we face the long‐term effects of the coronavirus disease 2019 (COVID‐19), we anticipate that the need for patient support post discharge will increase. Added to what has been noted as the effects of posthospital syndrome are the postillness effects that have been specifically associated with COVID‐19, which include decreased ejection fraction, pulmonary changes, posttraumatic stress, and persistent paresthesia.^47^ The older population who is already challenged with employing strategies to increase resilience and maintain independence may now have added challenges to overcome after discharge if they were admitted with COVID‐19. An additional result of the pandemic that could negatively affect instrumental support may be a hesitancy for people willing to reach out to provide help or ask for help from others. This may be directly related to limitations on social interactions based on recommendations by the healthcare officials to isolate, maintain physical distance, and only socialize with close family.^48^ Social interaction can take place via applications such as Microsoft Teams, Zoom, or FaceTime, but they cannot replace tangible assistance for personal care needs.

## Data Availability

Data sharing is not applicable to this article as no datasets were generated or analysed during the current study.
